# Supplementary material for: Allosteric modulation of the Lon protease via ssDNA binding and local charge changes
Source: J Biol Chem. 2024 Nov 13;301(1):107993. doi: 10.1016/j.jbc.2024.107993 (PMC11719849; doi:10.1016/j.jbc.2024.107993)
Supplement: Supporting information [file mmc1.docx]

**Supporting Information**

**
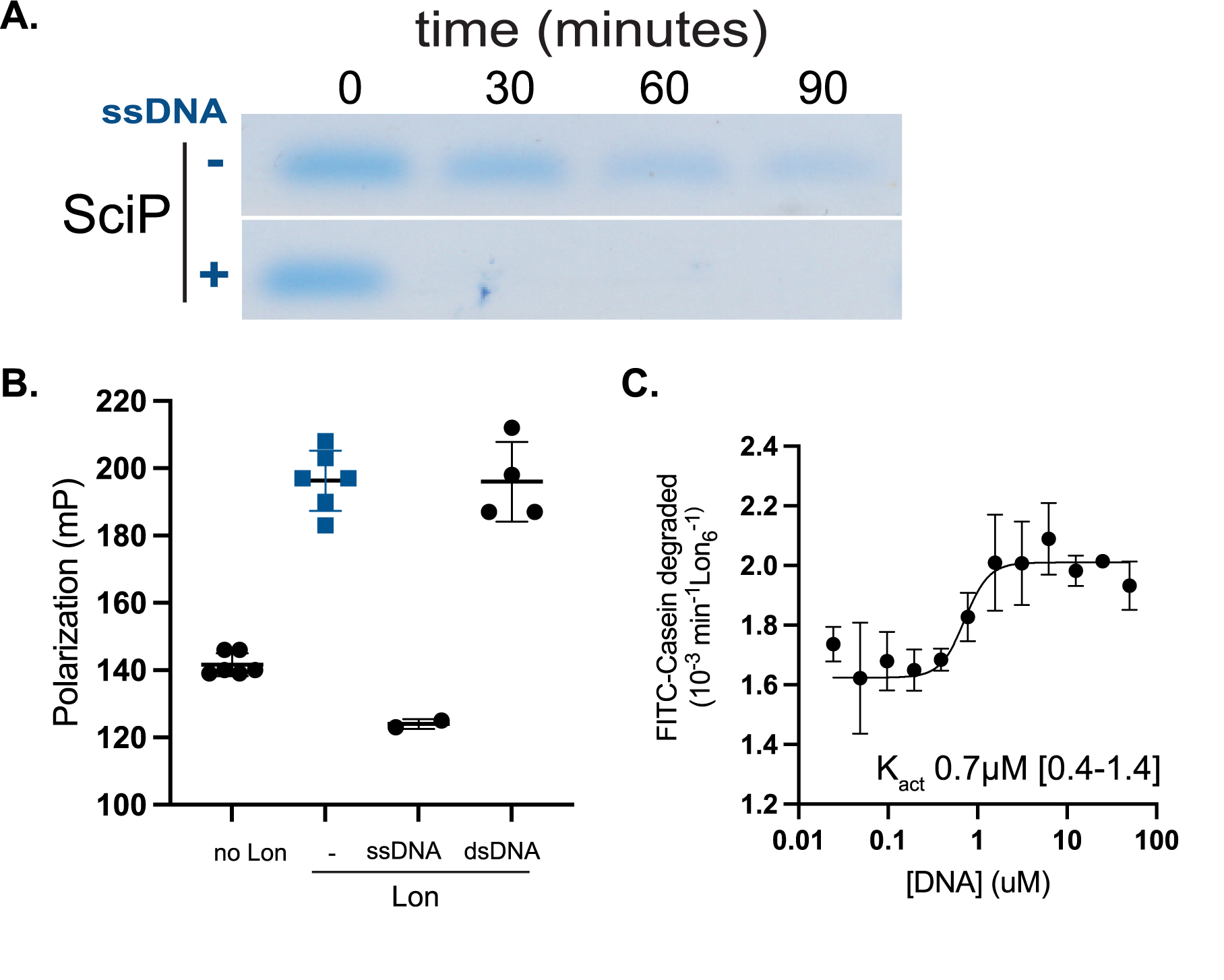
**

**Supporting information Figure 1.** A. *In vitro* degradation of SciP by Lon with and without ssDNA. Assay was performed using 0.1µM Lon, 20µM ssDNA and 5 µM SciP. B**.** Fluorescent polarization assays using FAM-ssDNA and competing non-fluorescent ssDNA and dsDNA. Lon (0.1µM) measured with 25nM ssDNA (n=2-6). C. *In vitro* degradation of 125µg/mL FITC-casein with titrating ssDNA (n=3). Error bars report the standard deviation.


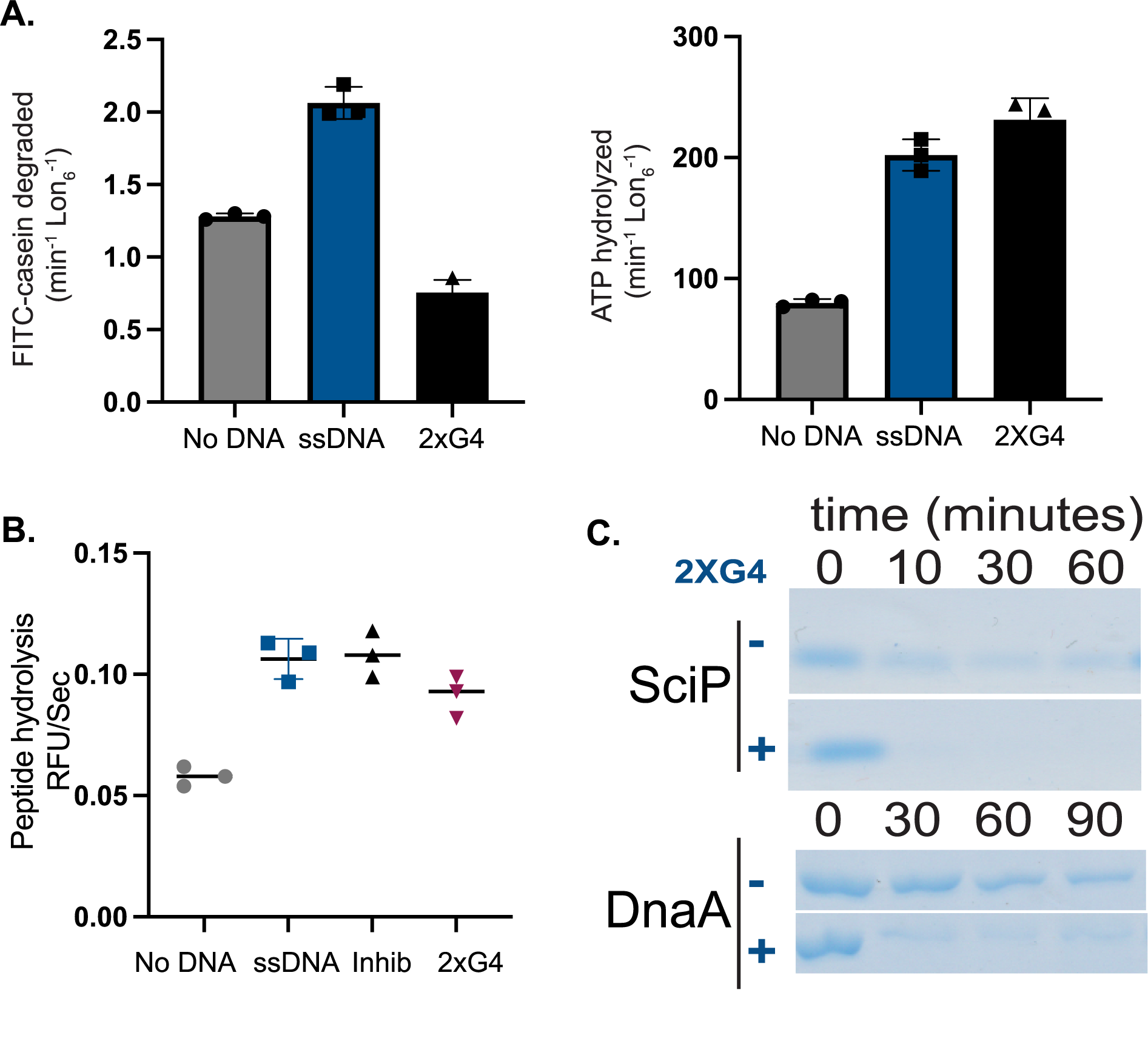


**Supporting Information Figure 2.** A**.** Dual *In vitro* degradation and ATP hydrolysis assays using ssDNA with and without ssDNA containing 2 G4 DNA sequences (2XG4) inserted (n=3). B. Peptide hydrolysis by Lon alone or with various ssDNA species with125µM fluor-peptide and an ATP regeneration mix, 2mM ATP, Creatine Kinase, Creatine phosphate and 125µg/mL casein. Inhib DNA contains two G quadraplexes (n=3). C. Gel based *in vitro* degradation of SciP (5µM) and DnaA (5µM) by 2XG4 ssDNA(20µM) (assay described in Figure 1). Error bars represent the standard deviation.

**
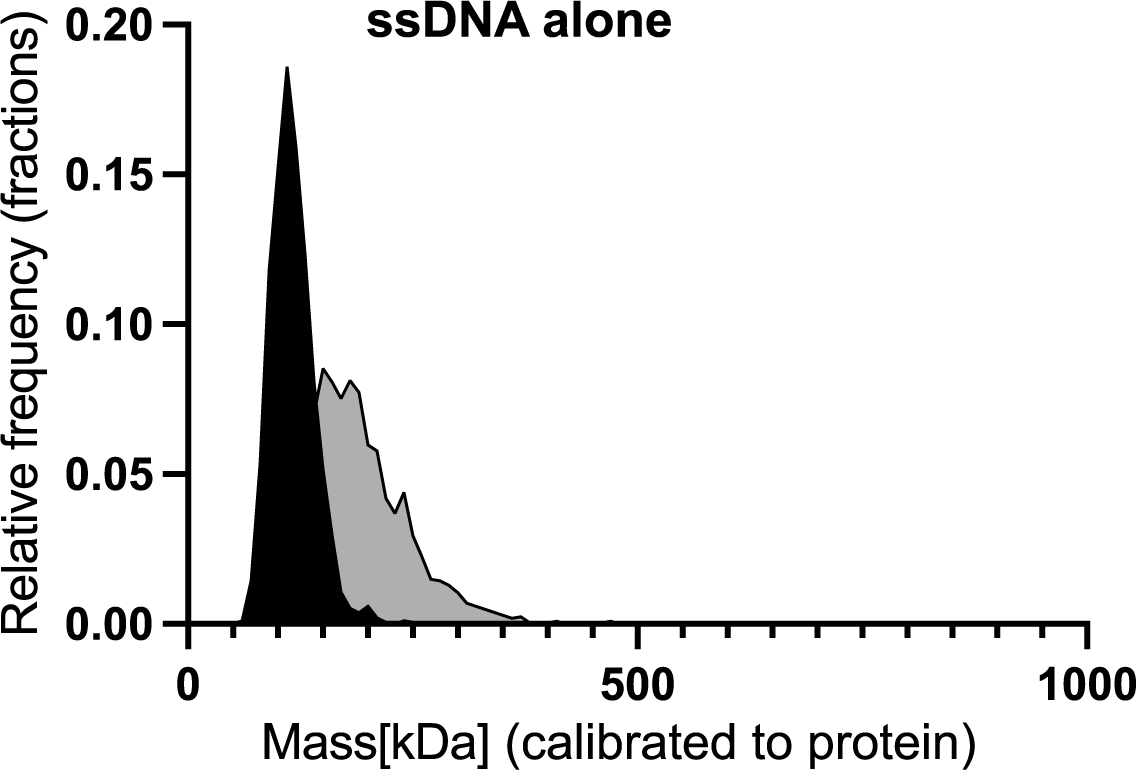
**

**Supporting Information Figure 3. ssDNA alone does not show high molecular weight species.** Mass photometry of ssDNA (20µM) alone (described in Figure 2). No mass can be assigned to ssDNA alone.

**
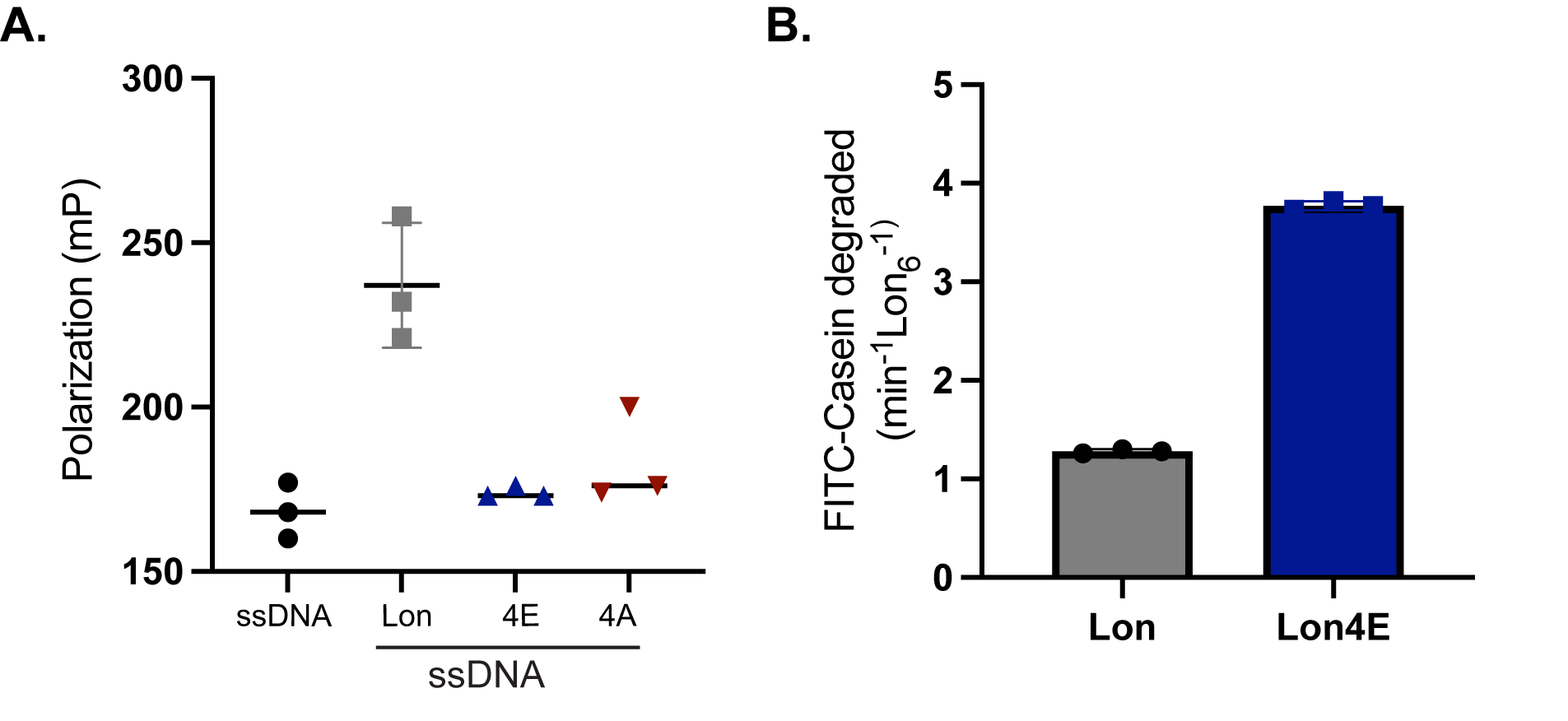
**

**Supporting Information Figure 4.**  **Lon4E and Lon4A do not bind to DNA, but Lon4E increases proteolysis**. A. Fluorescent polarization with FAM-ssDNA and Lon variants. 0.1µM Lon variant measured with 25nM ssDNA (n=3). B. *In vitro* degradation of FITC-casein by Lon or Lon4E (described Figure 2) (n=3). Error bars represent the standard deviation.

**
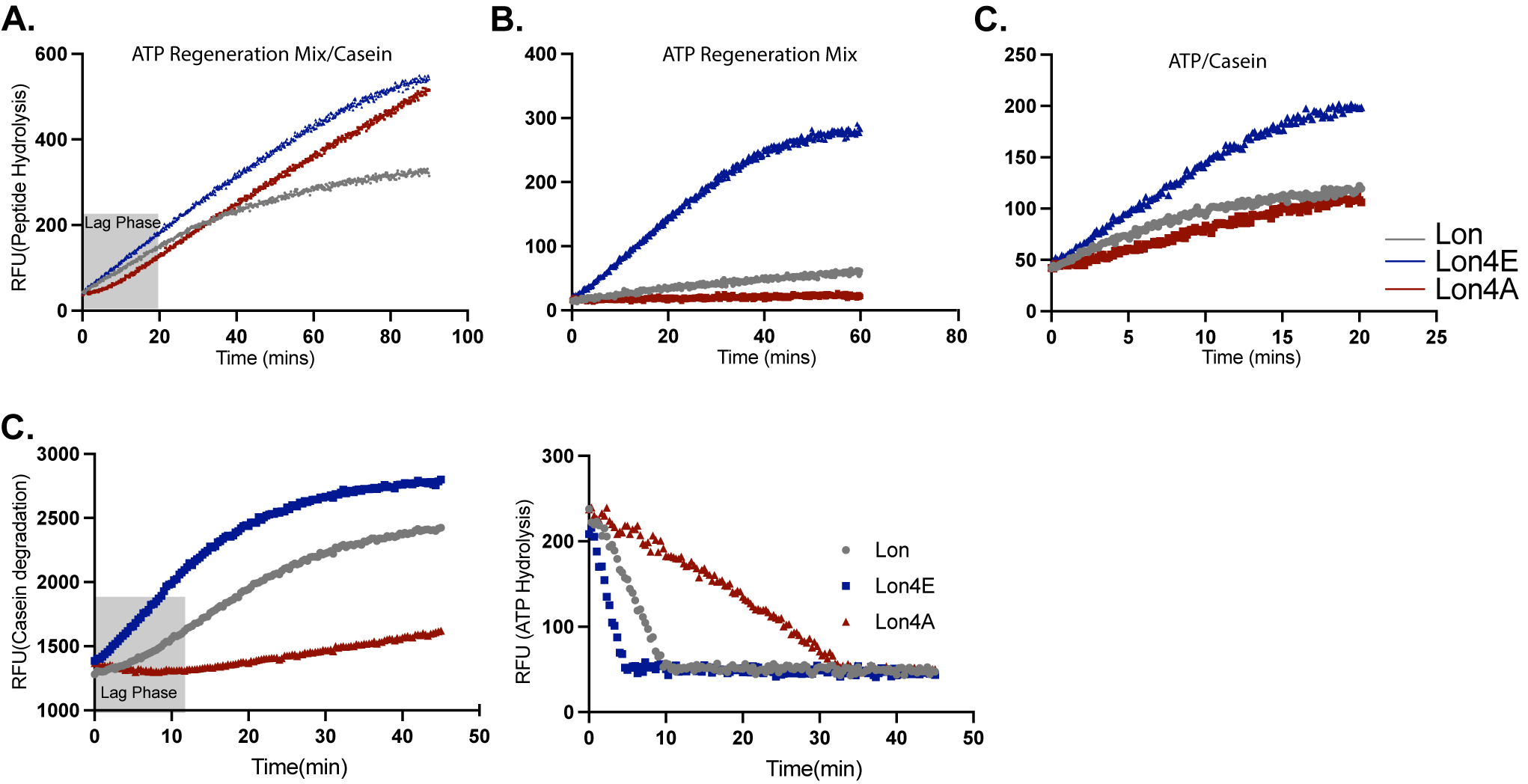
**

**Supporting Information Figure 5. All Lon variants hydrolyze peptides with substate and ATP, but the Lon4A has a lag period.** A. Peptide hydrolysis by Lon variants of 125µM fluor-peptide and an ATP regeneration mix, 2mM ATP, Creatine Kinase, Creatine phosphate and 125µg/mL casein. B. Peptidase activity using an ATP regeneration mix without the addition of Casein. C. Peptidase activity with 2mM ATP (not ATP regeneration components) and 125µg/mL Casein. D Raw curves of dual *in vitro* proteolysis and ATP hydrolysis by Lon variants. Grey box defines the lag phase for Lon4A during peptide hydrolysis and proteolysis. All assays were an n=3 with one replicate shown.


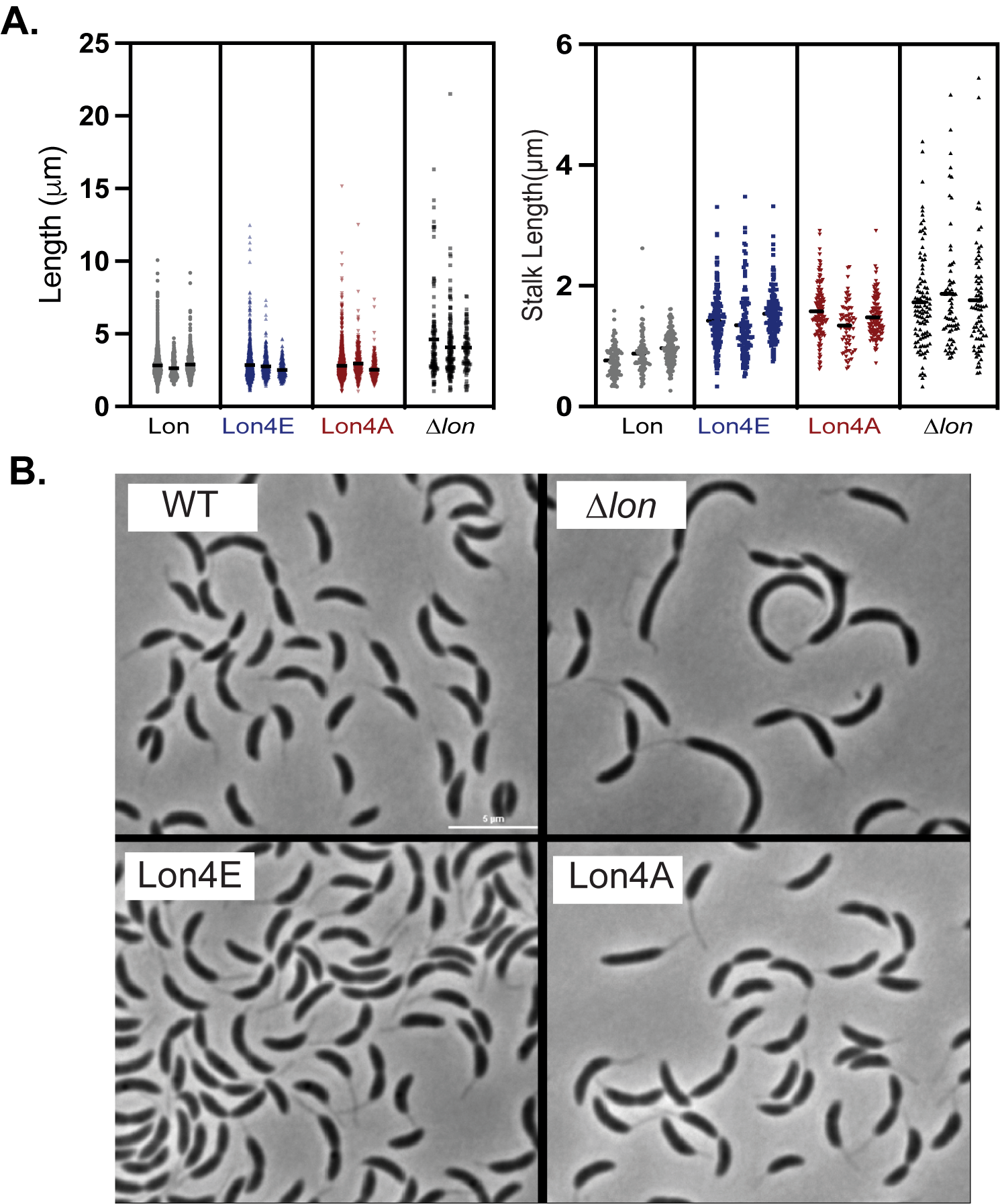


**Supporting Information Figure 6. Lon4E and Lon4A have similar cell lengths but exhibit longer stalks.** A**.** Cell length (MicrobeJ, Fiji) and stalk length measurements (Fiji) of the Lon alleles with n=3. B. Representative phase contrast microscopy of exponentially growing cells using 100X oil immersion (scale bar is 5µM).

**
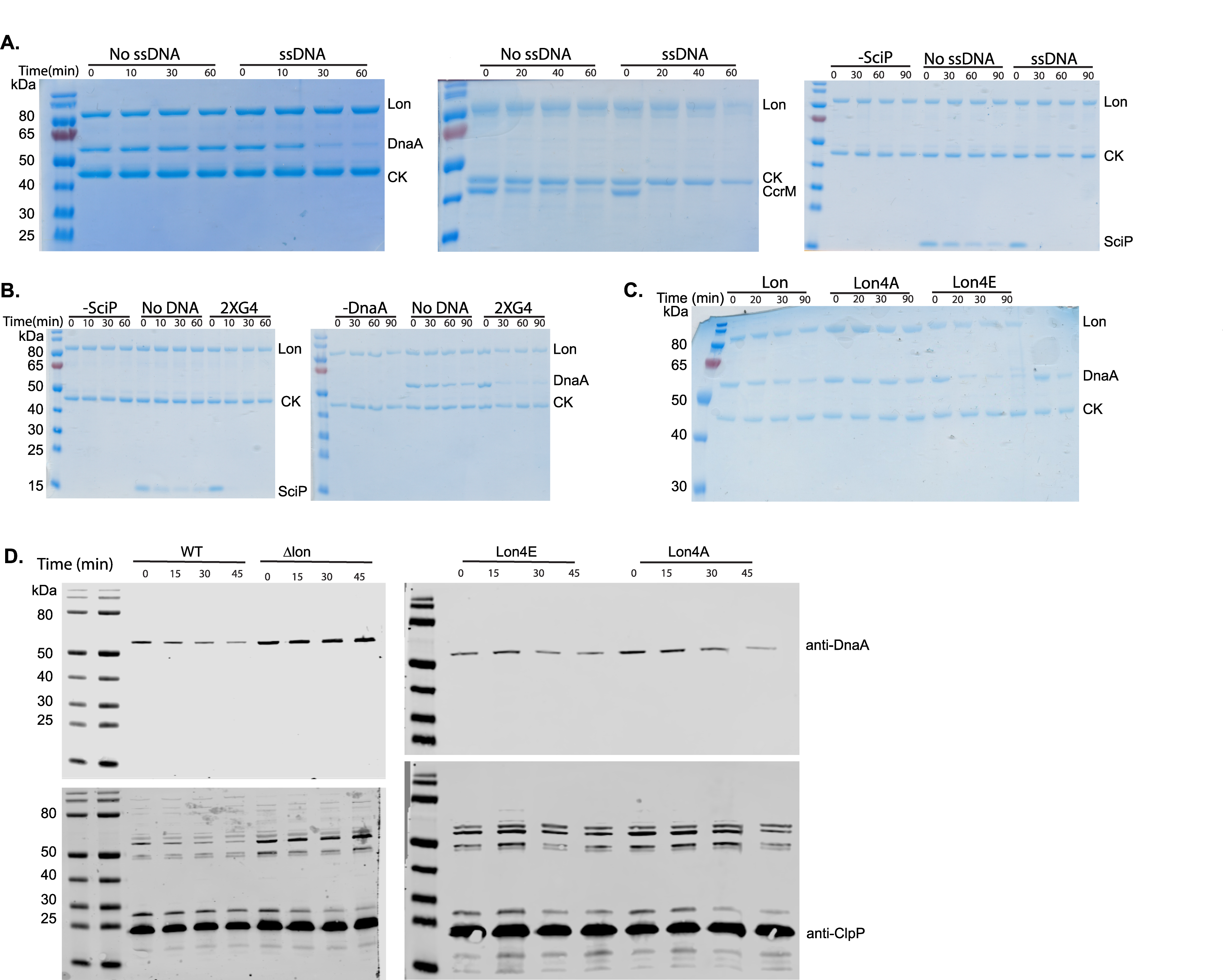
**

**Supporting Information Figure 7.** A. Original gels of *in vitro* degradation assays for Figure 1 and S1. B. Figure S2. C. Figure 3. D. Original western blots of translational shutoff experiments Figure 3

**Supplemental Table 1. Plasmids and cell strains used in this study.**

| **Plasmid or strain** | **Relevant characteristics** | **Comments** | **Reference** |
| --- | --- | --- | --- |
| CPC176 | Wild type strain | NA1000 |  |
| CPC667 | ∆*lon* | Clean delete | Lab collection |
| CPC741 | Lon4E | Allele replacement pNPTS-138 plasmid | (1) |
| CPC1278 | Lon4A | Allele replacement pNPTS-138 plasmid for Lon4A strain | This study |
| CPC103 | ∆lon:spec |  | (2) |
| EPC1590 | pBAD33-His_6_sumo-DnaA | BL21(DE3) Chloramphenicol | (3) |
| EPC446 | pET23b-His_6_sumo-CcrM | Bl21(DE3) Ampicillin | Lab collection |
| EPC565 | 375 SciP | Bl21(DE3) Ampicillin | Lab collection |
| EPC460 | pBAD33-Lon | BL21(DE3) Chloramphenicol | Lab collection |
| EPC1504 | pBAD33-Lon4E | Top10 Chloramphenicol | (1) |
| EPC1796 | pBAD33-Lon4A | BL21(DE3) Chloramphenicol | This study |

**Supporting Information Table 2. DNA sequences used in this study.**

| **Name** | **DNA Sequence** |
| --- | --- |
| OPC698-Stim | 5’TCGATTCTCGAGTTAGTCGTCTTCTGGTGCCGGAAA3’ |
| OPC498-Inhib | TGGGGTTAACGCTCTGTTAATCATGGGGAT |
| OJO19 (2XG4) | TCGGGGCTCGAGTTAGTCGTCTTCTGGTGGGGGAAA |
| FAM-ssDNA | FAM-AACGGATGATCCACAGGAGAGTCTGGCGCAGGGCGAGAG |
| G1Box | AACGGATGATCCACAGGAGAGTCTGGCGCAGGGCGAGAG |
| G1Box Reverse complement | CTCTCGCCCTGCGCCAGACTCTCCTGTGGATCATCCGTT |
| Lon4A_pNPTS | ACCGCAGCCATCGACCTCGTCGAGAGCGA |
| Lon4A_pNPTS | TGCGGCAGCGCCCCACGGGATCGACAGCAG |
| Lon4A_pBAD | ATCCCGTGGGGCGCTGCCGCAACCGCAGCCATCGACCTCG |
| Lon4A_pBAD | CGAGGTCGATGGCTGCGGTTGCGGCAGCGCCCCACGGGAT |

1. Zeinert, R. D., Ogdahl, J. L., Liu, J., Yang, Q., Du, Y., Barros, B. B., Freddolino, P. L., Haynes, C. M., and Chien, P. (2022) A legacy role for DNA binding of Lon protects against genotoxic stress. *bioRxiv*. doi:10.1101/317677

2. Wright, R., Stephens, C., Zweiger, G., Shapiro, L., and Alley, M. R. (1996) Caulobacter Lon protease has a critical role in cell-cycle control of DNA methylation. *Genes Dev.* **10**, 1532–1542

3. Liu, J., Zeinert, R., Francis, L., and Chien, P. (2019) Lon recognition of the replication initiator DnaA requires a bipartite degron. *Mol. Microbiol.* **111**, 176–186
